# A Case of Pancreatic Cyst1Read at the thirty-ninth annual meeting of the Medical Association of Central New York, at Syracuse, October 16, 1906.

**Published:** 1907-06

**Authors:** William D. Johnson

**Affiliations:** Batavia, N.Y.


### A Case of Pancreatic Cyst.[1]

By WILLIAM D. JOHNSON, M.D., Batavia, N.Y.

I AM prompted to report this case of necrosis and cyst of the pancreas at this time, because of the growing interest in diseases of the pancreas in general and pancreatic cyst, in particular, as the one disease of this organ amenable to safe surgical treatment followed by recovery in a large percentage of cases.

I am also influenced by a recognition of the fact that when a sufficient number of case histories have been recorded an intelligent sifting of the symptoms and antemortem findings, will result in formulating for this disease a rational diagnostic formula that will lead to more preoperative diagnosis than one in five, as at present.

The patient was a woman, aged thirty, married, with a negative family history, as regards this disease. She had scarlet fever during childhood and grippe when seventeen, followed by ill health for about six months. For eighteen months previous to the onset of the present illness she had some digestive disorder which was gradually progressive. Six months before the onset, she suffered from a well marked attack of hepatic colic and in the succeeding months had three other rather severe attacks of the same nature, the history of which is quite typical of the passage of gallstones. During these attacks she was slightly jaundiced, the skin not regaining a normal color at any time. No gallstones were discovered or searched for. There was all this time tenderness in the epigastric region, variable gaseous distention, eructations, occasional nausea, but no vomiting, and during the intervals between the acute attacks, she was able to do her housework.

She was seized with an attack July 21, 1905, more severe than the previous ones but similar in kind, and for the first time she vomited at the onset. The pain radiated upward and into both shoulders and was intermittent and colicky in character at first, but later became continuous. Morphine had little power to overcome it. Profuse salivation was present during the first two

1. Read at the thirty-ninth annual meeting of the Medical Association of Central New York, at Syracuse, October 16, 1906.

days. After this, it was absent or lost sight of in the presence of more urgent symptoms, the pain becoming constant and the vomiting nearly so, no food being retained for nearly a week, and the patient says she did not sleep for ten days. After this, the symptoms began to abate somewhat, and at the end of two weeks for the first time an epigastric fulness began to be noticed. This fulness gradually increased and caused only a sense of pressure but no acute pain.

I first saw her in consultation with Dr. Prince six weeks after the onset, and at that time the tumor in the upper left quadrant of the abdomen was causing all the discomfort she had. She had lost thirty pounds in weight and was very weak. The skin had a dirty brownish tinge but typical jaundice was absent. There was slight fever, pulse 120, occasional vomiting, the stomach being capable of holding only a few ounces at a time. Constipation was present. The urine contained neither albumin nor sugar. Respiration was impeded by the fulness interfering with the action of the diaphragm. The tumor presenting in the epigastric region was globular and very tense, the fluctuation waves being short and sharp as of a fluid under pressure. Percussion showed the stomach and colon displaced to the level of the umbilicus. The heart was displaced upward and outward, the apex being in the fourth interspace outside the nipple line. The area of dulness from the fluid, extended to the left and down below the region of the kidney and there was slight bulging in the costovertebral angle. The free border of the ribs was elevated and there was no descent of the tumor on inspiration. The history of the case and the examination led me to at once make a diagnosis of pancreatic cyst and advise opening and draining of the same.

Three days later, preparations having been made to operate at her home, I went there and was much surprised to find that during the night the tumor had disappeared entirely. She said that following a sensation of something tearing in the epigastric region, there had been a gradual subsidence of the swelling and relief of the pressure and discomfort.

The fluid, which had almost certainly escaped through the foramen of Winslow, was easily detected in the greater peritoneal cavity, presenting the usual signs of ascites, and was causing absolutely no symptoms of irritation or septic intoxication. In view of this emptying of the lesser peritoneal cavity and the relief of symptoms we decided to await further developments.

The fluid was absorbed in a few days, this absorption being accompanied by profuse diruesis, 80 ounces in twenty-four hours. The patient continued to improve, sat up and was entirely comfortable for a few days. But the signs of a refilling of the lesser peritoneal cavity soon appeared and at the end of twenty-five days from the emptying of the sac there was as great distention as before; and in the meantime, the patient having entered the Batavia Hospital October 2, I operated, opening the abdomen above the umbilicus in the middle line. The gastrohepatic omentum presented in the opening and so tightly stretched was it that, on introducing a trocar, there occurred a large stellate tear allowing free egress of the thin coffee colored contents. The gall bladder seemed normal and contained one large calculus. The foramen of Winslow having closed by plastic adhesion, the margins of the cyst wall were sewn to the abdominal wall and the cavity explored. Directly at the back lay the pancreas, necrosed, with large loose slaty colored fragments of its body, some free but most of them loosely attached, spread out over the floor of the cavity. The wall of the cyst was smooth, shiny, red gray in color and could not be separated from the surrounding structures. At the opening the wall was one-fourth of an inch thick and somewhat easily torn. A large drainage tube was placed deep in the cavity and the external wound partly closed.

The cyst contained four liters of thin coffee colored fluid, which on standing was covered by a thin oily looking film. It was odorless, alkaline in reaction and had a sp. gr. of 1009. Albumin was abundant, glucose and ferments absent. It contained a few red blood cells, some fatty granular cells and necrotic tissue. There was no leucin, tyrosin or cholesterin and no pus. On culture it was found to be sterile. Blood examination showed four million eight hundred thousand reds, seven thousand whites, differential count of which gave

| | |
|---|---|
| Polymorphoneuclear | 70% |
| Small nononeuclear | 17% |
| Large nononeuclear | 10% |
| Eosinophiles | 1% |
| Transitional | 2% |

For the very careful examination of the fluid and blood I am indebted to Dr. Cottis.

The patient stood the operation well and began to improve, this improvement continuing for about a week. Large masses of the necrotic pancreas came away and one of these fragments obstructing the narrowed outlet, caused the secretion of the remaining portions of healthy pancreatic tissue to be retained under pressure. About this time the fluid, which at first was inert began to acquire digestive power irritating the skin severely. There occurred for the first time fat necrosis of the perirenal fat and severe intoxication, with fever, etc., and her condition became grave. A large swelling occurred in the region of the left kidney and just about as an opening in the loin was decided upon, a free communication with the drainage opening in the epigastric region occurred, accompanied by a discharge of necrosed fat masses and pus. A large flexible tube was used to irrigate this cavity, it being possible to pass it down and to the left at least ten inches. From this time on, she made a rapid recovery and left the hospital October 23, three weeks from the time of entering. A sinus still persists from which a small amount of fluid drains, the fluid being inert. Repeated efforts have been made to promote closure of this sinus, all of which have been ineffectual.

The points of interest in this case are:

1. The occurrence of necrosis and pancreatic cyst following hepatic colic.

2. The closure of the foramen of Winslow and retention of fluid in the lesser peritoneal cavity, and the opening under pressure of this foramen with discharge of the fluid into the greater cavity; its absorption and elimination without producing serious symptoms; the subsequent closure and refilling.

3. The resumption of digestive activity of the fluid after relief of pressure and separation of the necrosed portion of the pancreas.

The etiology and pathology of pancreatic cysts are, at present, not well understood, and even in the classification there is much confusion. Tilger thinks all cysts except those connected with new growths, depend upon hindrance to the outflow of the secretion of the gland and consequent degeneration of gland epithelium, which permit autodigestion and escape of secretion.

The association of gallstones in this case and the history of their passage point to a probable obstruction of the duct of Wirsung. At the same time the presence of a gallstone in the common duct, which passes in the margin of the foramen of Winslow, may have set up a low grade inflammation which closed the passage from the lesser to the greater peritoneal cavity, the fluid being retained in the lesser sac. If gallstones were a common cause of obstruction of the pancreatic duct and consequent formation of pancreatic cysts, women should be the most frequent sufferers and most cases should occur after the fortieth year, but the sexes are equally affected and most cases occur before this age, fifty per cent. between thirty and forty. Acute pancreatitis likewise cannot be a frequent cause as 90% of the cases occur in men at a later period of life.

Gangrene of the pancreas is followed by a collection of pus in the lesser peritoneal sac in some instances and is necessarily due to septic microorganisms. This case was one of aseptic necrosis as shown by the cultures taken at the time of operation and the nontoxic character of the fluid when liberated into the general peritoneal cavity, which occurred three weeks previous to the operation. In the embryo the pancreas springs as a bud from the duodenum and is at first entirely surrounded by peritoneum, the posterior mesopancreas later becomes absorbed and is replaced by areolar tissue which is continuous with the areolar tissue surrounding the left kidney. This connection accounts for the line of extension of fat necrosis subsequent to operation. That a portion of the pancreas remained intact is proven by the resumption of activity of the fluid discharged, and the absence of diabetes which would have occurred had the islands of Langerhans all been destroyed.

